# Mobile Ecological Tracking of mood as a predictor for resilience among male and female Israeli combatants

**DOI:** 10.1192/j.eurpsy.2022.609

**Published:** 2022-09-01

**Authors:** Y. Gilboa, M. Nahum

**Affiliations:** The Hebrew University of Jerusalem, School Of Occupational Therapy, Jerusalem, Israel

**Keywords:** Gender differences, ecological momentary assessments, QOL, Basic military training

## Abstract

**Introduction:**

Background: Mental resilience refers to the capacity to overcome the negative effects of setbacks and associated stress on performance. In the face of stressors, lack of mental resilience may even cause psychopathology, such as depression. While all combatants are exposed to stressors, female combatants face additional challenges compared with their male counterparts. Resilience is often measured using retrospective self-reports, which do not consider ecological fluctuations across situations and environments. A mobile ecological momentary monitoring allowed us to study gender differences in factors contributing to resilience.

**Objectives:**

Objective: We aimed to characterize gender differences in resilience trajectory in combatants using ecological momentary assessments (EMA).

**Methods:**

Methods: 156 Combatants (98F, 58M) completed mood EMA daily for two weeks using a mobile app. In addition, resilience, QOL and mental health questionnaires were administered three times in four weeks. Stepwise regression models were used to predict resilience after 2-4 weeks.

**Results:**

Results: Female combatants reported higher levels of anxiety and lower resilience, self efficacy and QOL, as well as higher mood variability over time (t(149)=4.9, p<.0001). In addition, while for females, baseline anxiety, self-efficacy and mood EMA all contributed to resilience prediction (37% of variance explained), baseline anxiety was the sole predictor for males (explaining 28% of variance).

**Conclusions:**

Conclusion: Gender differences in resilience were found in combatants who participate in the same occupation. These results emphasize the importance of considering the inclusion smartphone-delivered EMA tools in QOL models.

**Disclosure:**

No significant relationships.

